# Confidence-building in a collaborative, multidisciplinary mock page activity for fourth-year medical students

**DOI:** 10.1080/10872981.2023.2173995

**Published:** 2023-02-02

**Authors:** Melissa Victory Brodman, Aaleena Zaidi, Sidra Qureshi

**Affiliations:** a Internal Medicine resident, University of Texas Medical Branch (UTMB), Galveston, TX, USA; b John Sealy School of Medicine, University of Texas Medical Branch (UTMB), Galveston, TX, USA; c Department of Internal Medicine at UTMB, Transition to Residency Course Director at John Sealy School of Medicine, Galveston, TX, USA

**Keywords:** Transition to residency, interprofessional communication, medical school curriculum, medical education innovation, mock page

## Abstract

Graduating medical students require multiple skills that are not traditionally taught in medical school. Mock paging activities, where school of medicine (SOM) students receive pages as if from nursing staff in a practice environment, are being used as a tool to teach communication and to enhance confidence. Prior small-scale, specialty specific mock page activities have demonstrated improvement in confidence, communication with other healthcare personnel, and medical decision-making.^3-5^ This Mock Page Activity aimed to evaluate the effect on confidence and communication for large graduating SOM class entering different specialties. SOM faculty collaborated with the School of Nursing (SON) faculty in the design and implementation of this activity. Medical students completed pre-/post surveys regarding confidence in communication, interaction with other healthcare professionals, and patient management. Two years of medical student survey data (n=420) after the Mock Page Activity implementation demonstrated a significant increase in general confidence (30.4%, p <0.001) related to receiving communication from nursing staff, making clinical decisions over the phone, and using a pager as a communication device. This multidisciplinary Mock Page Activity improved communication and confidence during paging activities for a large group of medical students pursuing different specialties. Strengths of the study include being the largest, specialty non-specific mock page activity reported in the literature. Limitations include varied individual experience of students and questionable benefits for students pursuing non-patient care careers in medicine. Future directions include iterative improvement based on feedback and incorporation of an interprofessional debrief session to ensure equal benefit to the participating SON students.

## Introduction

Fourth-year medical school students are expected to achieve certain milestones by the start of internship, but research finds there are gaps in education that threaten a smooth transition [1]. The Association of American Medical Colleges (AAMC) has published guidelines to provide expectations of activities that all U.S. medical students should be able to perform upon entering residency, known as the Core Entrustable Professional Activities for Entering Residency [2]. These practical skills are not traditionally encompassed in medical school education, thus innovative curricula attempt to bridge gaps in education and fulfill Core EPAs in a standardized fashion. One such curricular innovation is a mock paging activity, which is a simulated encounter where medical students receive pages as if from nursing staff about fictitious patients similar to real-life hospital paging interactions. This creates a no-risk environment to practice communication, interprofessional collaboration, and clinical decision making. The learner feels free to make mistakes and ask questions, a luxury, which may not readily present itself once real patients are at stake. A mock paging activity fulfills the following AAMC Core EPAs: 1) gather a history and physical exam, 2) prioritize a differential, 4) enter and discuss orders and prescriptions, 9) collaborate as part of an interprofessional team, and 10) recognize urgent/emergent need for patient evaluation) [2]. Along with fulfilling Core EPAs, a mock paging activity has a multitude of other benefits. Surgery-specific mock-page activities have shown improvement in clinical decision-making as well as confidence after participation [3]. Surgery programs have created a standardized residency boot camp that incorporating a mock paging activity and hosted it at numerous Schools of Medicine across the United States. This surgery-specific national mock page study reported overall improved communication and clinical decision making over the four-week course among all participants. Other small-scale mock paging activities have reported improved confidence and communication skills from the experience [4,5]. One study evaluated the post-activity feedback that nurses gave to medical students and noted similarities between the activity and their real-life stories, suggesting that these paging activities reflect real-life scenarios [6]. Of equal importance, mock paging activities prepare medical students for the inherent interprofessional environment of health care [4,7]. In a mock paging pilot study between School of Medicine and School of Nursing students, both groups reported improved knowledge of the other’s role in health care delivery and ability to speak with other health care professionals after the activity and a debrief session [4]. These experiences enhance the participants’ understanding of how various professions contribute to patient care [4,5]. Mock paging activities previously reported in the literature have been included in courses aimed at preparing senior medical students for their intern year, such as a transition to residency or bootcamp style course [3–5]. To our knowledge, only one large-scale, multi-institutional mock paging activity has been published which took place with small groups at each location. Other published studies have represented smaller cohorts and/or were specialty specific. There is a need to evaluate the benefits of a mock paging activity designed to engage the entire graduating student body entering different specialties to evaluate if widespread improvement in confidence and communication is possible.

Our institution, like more than half of medical schools across the United States, offers a Transition to Residency (TTR) course [8]. This is a mandatory course for all graduating fourth-year medical students at the John Sealy School of Medicine at University of Texas Medical Branch. It is composed of various lectures, workshops, hands-on skill sessions, and simulations designed to prepare students in all specialties for residency. The goal of this course at our institution is for students to be able to construct a framework for transitioning from medical student to intern and to further develop within five domains: knowledge base, interpersonal and communication skills, culture of safety, personal and professional growth, and professionalism. Recognizing the multifaceted benefits of a mock paging activity, the TTR course leadership designed and implemented a required Mock Page Activity in collaboration with our institution’s School of Nursing (SON). Unlike other published mock paging activities, our activity was designed to encompass all specialties of fourth-year school of medicine (SOM) students. Similar to another study, our Mock Page Activity paired fourth-year medical students with the nursing senior students in their capstone course, thereby promoting interprofessional collaboration within two groups of graduating healthcare providers [4]. Utilizing pre- and post-surveys in both 2021 and 2022 implementation of the Mock Page Activity, we aimed to determine if a large-scale, specialty nonspecific mock paging activity increases confidence in communication, medical decision making, and paging interactions among fourth year SOM students.

## Methods

### Participants

In 2021, 236 fourth-year SOM students and, in 2022, 222 fourth-year SOM students were enrolled in the required Transition to Residency (TTR). All students participated in the Mock Page Activity during these 2 years of the course. Table 1 lists the pursued specialties of all students.

**Table 1.** Specialties pursued by graduating school of medicine students (*n* = 420, responses = 482) from Class of 2021 and Class of 2022. Disclaimer: this list included SOM students applying to more than one specialty and exceeds the total number of participants. Demographic data is otherwise not reported because it was not relevant to the statistical analysis.

Specialty	Number of applicants
Anesthesiology	44
Dermatology	11
Emergency Medicine	30
Family Medicine	41
General Surgery	28
Internal Medicine	91
Internal Medicine/Pediatrics	2
Interventional Radiology – Integrated	5
Neurology	5
Neurosurgery	6
OB/GYN	16
Ophthalmology	9
Orthopaedic Surgery	21
Otolaryngology	8
Pathology	9
Pediatrics	49
Plastic surgery	7
PM&R	6
Psychiatry	46
Radiation Oncology	1
Radiology-Diagnostic	24
Thoracic Surgery	1
Transitional Year	8
Urology	4
Vascular Surgery	3
Other	7
**Total**	**482**

### Mock paging activity design

The Mock Page Activity was incorporated into the length of the TTR course. Nine clinical patient cases were adapted from ‘Clinical Reasoning Cases in Nursing’ by Harding and Snyder [9]. These scenarios were selected to engage students across specialties and to prompt different depths of clinical decision making. Case writing was performed in collaboration with Nursing faculty to ensure each case had stable patient interactions labeled ‘Level A’, intermediate patient interactions labeled ‘Level B,’ and unstable and/or critical patient interactions labeled ‘Level C’. SOM students received a brief ‘check-out’ report with a patient list, while SON students had extensive information about the same patients to prepare SBAR (Situation-Background-Assessment-Recommendation) reports for each paging level interaction assigned to them. SOM students carried a physical pager during 1 week of the TTR course and were expected to respond promptly to pages received from SON students. During the paging interaction, the medical student listened intently to the SBAR report from the nursing student and was subsequently expected to ask questions to further extrapolate history then give verbal orders over the phone. Once medical students completed their week of ‘on-call’, they checked out their patient list and handed off their pager to the on-call medical students for the following week.

### Survey design

SOM students were asked to complete pre- and post-activity surveys. The Mock Page Activity pre-survey asked the following questions: 1) Overall, what concerns do you have regarding your upcoming role as physician-in-training? (free response), 2) Please rate your confidence level regarding your preparedness to engage in paging interactions: i) your ability to independently manage a patient over the phone, ii) your ability to use a pager as a communication method, iii) your ability to receive a SBAR report from nursing, and 3) Which of the following pager-related components are you most concerned with: i) Use of the pager, ii) Communicating with another care team member I have not previously interacted with, iii) Providing patient care without physically seeing a patient, and iv) None of the above. Answer choices for question 2 were on a 4-point Likert scale with 1 = not confident, 2 = somewhat confident, 3 = moderately confident, 4 = very confident. Multiple answer choices could be selected in question 3. On the post-survey question number two was asked identically. Question three was adjusted to ask, ‘After the Mock Page Activity, I feel more comfortable with … ’ with the aforementioned options.

### IRB statement

The Institutional Review Board reviewed the study and determined that this submission does not meet the definition of ‘human subject research’, as defined by the regulations at 45 CFR 46.102. Therefore, the project does not require IRB approval or oversight. Additionally, this project was approved by the School of Medicine’s Curriculum Committee for publication.

### Survey analysis

Pre- and post-survey question responses related to confidence were analyzed using two-tailed paired t-test statistics. Duplicate responses were found and only the first response was included. Descriptive statistics were used to evaluate these questions: ‘which of the following pager-related components are you most concerned with?’ and ‘after the Mock Page Activity, I feel more comfortable with the following:’. Inductive thematic analysis was used for the free-response question ‘overall, what concerns do you have about your upcoming role as a physician in training.’ All authors reviewed the responses and discussed common themes, and then a coding system was made for responses. Coded themes included transition, clinical decision-making, work–life balance, setting (location, health record, technology), confidence, surgical skills, communication, and miscellaneous. Responses were coded and then reviewed by co-authors. Representative quotes were included.

## Results

During year 1, 220 of 236 (93%) SOM students completed both the pre- and post-survey. In the second year, 200 of 222 (90%) SOM students completed both surveys for a total of 420 responses used for analysis.

### Concerns as physician-in-training

When asked the question ‘Overall, what concerns do you have about your upcoming role as a physician in training?’, the most frequently expressed theme related to the nervous anticipation of the transition to internship, including issues with time management, patient load, independence, and responsibility. For example, one student wrote, ‘I don’t feel like I know enough to be a doctor with responsibilities,’ and another relayed concerns with, ‘having greater responsibility in patient care, having the confidence and skills to make decisions with more autonomy.’ Someone expressed, ‘It is a very different burden of responsibility level. When your job is to be a student, there’s a safety net. Now, our job is to be doctors. COVID really impacted our clinical training, and I have been out so long for interview season. I’m concerned about forgetting everything also!’

The second most frequent theme was clinical decision making. A student reports concerns with, ‘being “up-to-speed” as internship begins in terms of medical knowledge/clinical competency and medical decision making.’ Multiple students expressed deficit in knowledge leading to mistakes. For example, one student was concerned with ‘more responsibility and potentially making mistakes. My upcoming role is a little outside my comfort zone, but I am confident in my knowledge gained through school and my ability to learn quickly.’

### Confidence measures

**Figure 1.**
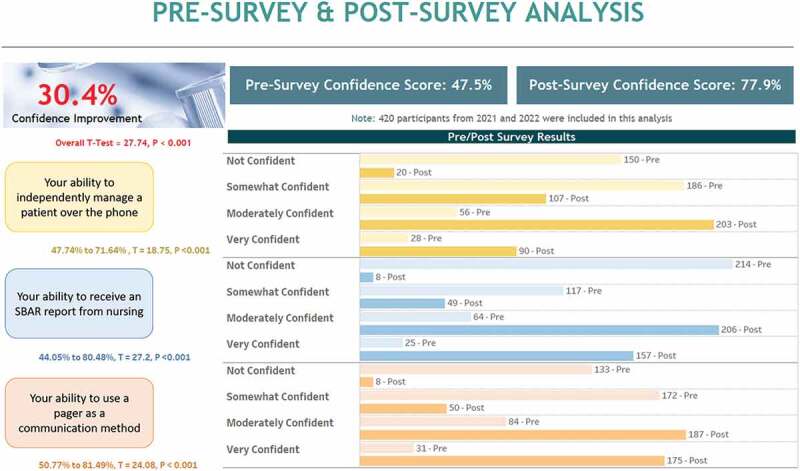
This figure illustrates the responses of 420 students in 2021 and 2022 during the Transition to Residency course when asked in both pre and post survey questions ‘Please rate your confidence level regarding your preparedness to engage in paging interactions as below’ with students being asked to rank the following questions on a 4 point Likert scale (1=not confident, 4=very confident): Your ability to manage a patient over the phone, Your ability to receive an SBAR from nursing, and Your ability to use a pager as a communication method. Each question when asked after the students went through the mock paging activity shows a significant increase in confidence.

Table 2 lists the statistics for each question pre- and post-mock page activity in each year as well as the combined 2 years of data. Medical students were asked in both pre- and post-surveys to rank on a 4-point Likert scale (1=not confident, 4=very confident) ‘Please rate your confidence level regarding your preparedness to engage in paging interactions as below’: Your ability to manage a patient over the phone, Your ability to receive an SBAR from nursing, and Your ability to use a pager as a communication method. Pre-activity confidence levels across both years and combined were near ‘somewhat confident’ (rated as 2). Post-activity confidence levels across both years and combined were near to or above ‘mostly confident’ (rated as 3). Increase in confidence was statistically significant for all questions for individual and combined years. Overall, students had a significant increase in confidence by 30.4% (*p* < 0.001), moving from somewhat (47.5%) to mostly confident (77.9%). This is visually represented in Figure 1.

**Table 2.** Descriptive statistics of pre-/post-activity confidence measures reported by graduating school of medicine students (*n* = 420) from Class of 2021 and Class of 2022. Confidence measured on a Likert scale (1=not confident, 4=very confident).

	Year One Mean (Variance)			Year Two Mean (Variance)			Combined		
	Pre-	Post-	*p*-value	Pre-	Post	p-value	Pre-	Post-	p-value
Use of pager	2.05 (.8)	3.18 (0.6)	<0.001	2.01 (0.8)	3.3 (0.4)	<0.001	2.0 (0.8)	3.25 (0.5)	<0.001
Receive an SBAR	1.8 (0.9)	3.15 (0.6)	<0.001	1.71 (0.7)	3.29 (0.4)	<0.001	1.7 (0.8)	3.2 (0.5)	<0.001
Manage a patient over phone	1.96 (0.8)	2.87 (0.6)	<0.001	1.85 (0.6)	2.85 (0.6)	<0.001	1.9 (0.7)	2.8 (0.6)	<0.001
Overall confidence	1.94 (0.6)	2.97 (0.5)	<0.001	1.86 (0.5)	3.0 (0.5)	<0.001	1.9 (0.6)	2.98 (0.5)	<0.001

### Mock page activity concerns to comfort level

Students were asked in a pre-survey question prior to the Mock Page Activity, ‘Which of the following pager-related components are you most concerned with?’ After the Mock Page Activity, the question was asked differently ‘Complete the following statement: After the Mock Page Activity, I feel more comfortable with the following.’ Answer choices for both pre- and post-survey questions had identical domains: Use of the pager itself, communicating with another care team member I have not previously interacted with, providing patient care without physically seeing a patient, and none of the above/other.

**Figure 2.**
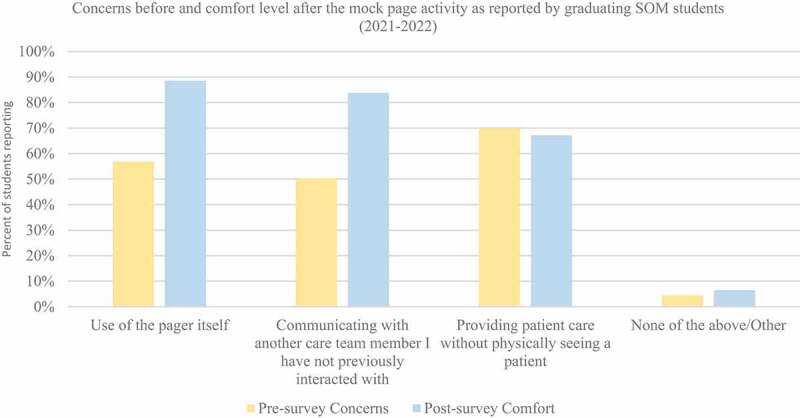
This figure illustrates the responses of 420 students in 2021 and 2022 during the Transition to Residency course when asked in a pre-survey question prior to the mock paging activity ‘Which of the following pager-related components are you most concerned with?’ After the mock paging activity, the question was asked differently ‘Complete the following statement: After the mock page activity, I feel more comfortable with the following.’ Answer choices for both pre and post survey questions were identical: Use of the pager itself, communicating with another care team member I have not previously interacted with, providing patient care without physically seeing a patient, and none of the above/other. For each domain comfort developed to a level that was equal or greater to the prior concern.

At least half of all students reported concerns with at least one of the domains, and multiple students reported concerns with 2 or more domains (Figure 2). 56% of students had concern regarding the use of the pager, but afterward, 88% of students felt more comfortable. 50% of students reported concerns with talking to new care team members, and after the activity, 83% felt more comfortable in such interactions. 70% of students had concerns with caring for patients without physically seeing them; whereas 67% of students felt more comfortable after the activity. For each domain, comfort developed to a level that was equivalent to or greater than the prior concern after the Mock Page Activity regarding the new responsibilities they will have as physicians-in-training. An example of SOM students who responded with other concerns reported, ‘None (I will not be using a pager in pathology)’ and ‘not directly pertinent to my specialty.’

## Discussion

Similar to prior small, specialty-specific mock page activities [3,4], our intervention shows that a large-scale, cross-specialty mock paging activity improves the confidence level and communication during paging interactions across a large group of graduating SOM student pursuing different specialties. A mock paging activity is an effective way to teach skills necessary to function during residency that may not have been taught in the basic science or clinical rotations of medical school.

Inductive thematic analysis of the SOM student concerns for their upcoming new role as physician-in-training suggest that students have significant anticipation regarding the transition to physician-in-training and the increased autonomy and impact of their decisions on individual patients. The implementation of the Mock Page Activity within the TTR course attempts to address some of the concerns voiced in the free response by students, which includes but is not limited to concerns with clinical decision making, autonomy, communication, and confidence. Through clinical paging interactions, students were allowed a safe space to act as a physician-in-training and practice autonomous clinical decision making.

Over the 2 years of the Mock Page Activity, we have seen a statistically significant increase in self-reported confidence in all domains, inferring that medical students were subsequently better prepared for paging interactions after going through this activity. The independent format of this activity emphasized the autonomy physicians-in-training will have during their training and improved their comfort regarding these interactions. Through this initiative, we have seen that the Mock Page Activity has been well received by SOM students, demonstrating the value of this activity for our large population of graduating SOM students.

Further, the Mock Page Activity embraced the interprofessional collaboration SOM students will encounter as interns by working with the senior SON students. The SOM students had a dramatic increase in comfort speaking with another care team member with whom they may not have spoken before. These simulated encounters of common intern-year experiences demonstrate benefit for most students across our two-year cohort. Of note, our students pursuing pathology did feel this specific activity and the entire course had limited utility for them as evidenced in the free-response questions. Further curricula development can investigate alternate experiences that may benefit this subsection of students.

Strengths of this study are the robust number of participants, diverse patient cases including all specialties, data of two consecutive years of activity implementation, the multidisciplinary nature of this activity, and the reproducibility of this curriculum. A limitation of this study includes being a single-center study and lack of follow-up data from physicians-in-training after the start of residency. Student feedback demonstrated the limited utility of such an activity for indirect patient care specialties, i.e., pathology. SOM student experience was affected by the individual SON students’ preparedness (i.e., self-creation of SBAR based on clinical data) and unpredictable nature of using pager technology (i.e., receiving incorrect call-back numbers, receiving actual hospital pages due to pager previous assignment or similarity to another numbers). Another limitation of this study is the lack of nursing student data and surveys that encompassed both schools to ensure mutual benefit.

Future direction of this activity includes further collaboration with School of Nursing to implement interprofessional objectives for the Mock Page Activity and align pre- and post-surveys for both SOM and SON students to determine if this collaborative Mock Page Activity has mutual benefit. Incorporation of debrief sessions would create space for self-reflection and perspective taking on how the nursing student perceived the paging encounter. Each school plans to incorporate student champions who will be part of the Mock Page Activity planning process and subsequent debrief. These champions will then summarize the debrief to their colleagues at an end-of-course meeting. Ensuring both SOM and SON students find value and benefit from this activity is critical to its longevity and sustainability as an interprofessional activity in both school’s curricula.
